# Evidence for an Association between Macular Degeneration and Thyroid Cancer in the Aged Population

**DOI:** 10.3390/ijerph15050902

**Published:** 2018-05-03

**Authors:** Shih-Yi Lin, Wu-Huei Hsu, Cheng-Li Lin, Cheng-Chieh Lin, Jane-Ming Lin, Yun-Lun Chang, Chung-Y. Hsu, Chia-Hung Kao

**Affiliations:** 1 Graduate Institute of Clinical Medical Science, College of Medicine, China Medical University, Taichung 40402, Taiwan; oasisbestonly@yahoo.com.tw (S.-Y.L.); Hsuwh@mail.cmuh.org.tw (W.-H.H.); ccling@mail.cmu.edu.tw (C.-C.L.); hsuc@mail.cmuh.org.tw (C.-Y.H.); 2 Division of Nephrology and Kidney Institute, China Medical University Hospital, Taichung 40447, Taiwan; mcspaghetti@gmail.com; 3 Department of Chest Medicine, China Medical University Hospital, Taichung 40447, Taiwan; 4 Management Office for Health Data, China Medical University Hospital, Taichung 40447, Taiwan; orangechengli@gmail.com; 5 College of Medicine, China Medical University, Taichung 40447, Taiwan; 6 Department of Family Medicine, China Medical University Hospital, Taichung 40447, Taiwan; 7 Department of Ophthalmology, China Medical University Hospital, Taichung 40447, Taiwan; D4301@mail.cmuh.tw; 8 Department of Nuclear Medicine and PET Center, China Medical University Hospital, Taichung 40447, Taiwan; 9 Department of Bioinformatics and Medical Engineering, Asia University, Taichung 40447, Taiwan

**Keywords:** thyroid cancer, age-related macular degeneration, national health insurance research database

## Abstract

Direct evidence of whether thyroid cancer patients have a higher risk of age-related macular degeneration (AMD) has yet to be investigated. Patients older than 50 years-old and newly diagnosed with thyroid cancer between 2000 and 2008 were identified from the national health insurance research database (NHIRD). We applied time-varying Cox proportional hazard models to assess the association between thyroid cancer and AMD. The multivariable models included conventional cardiovascular risk factors, myopia, vitreous floaters, hypothyroidism, hyperthyroidism, and treatment modality of thyroid cancer. The analysis process was stratified by age, gender, and comorbidity. In this study, 5253 patients were included in a thyroid cancer cohort (men 24.5%; median age 59.1 years (53.7–67.4 years), and 21,012 matched controls were included in a non-thyroid cancer cohort. The AMD incidence was 40.7 per 10,000 person/year in the thyroid cancer cohort. The thyroid cancer cohort had a higher risk (adjusted hazard ratio (aHR) = 1.38, 95% confidence interval, CI = 1.09–1.75) of AMD than the non-thyroid cohort. Thyroid cancer patients had a higher risk of AMD, especially the male patients (aHR = 1.92, 95% CI = 1.38–3.14) and the patients with comorbidities (aHR = 1.38, 95% CI = 1.09–1.74). In conclusion, thyroid cancer patients older than 50 years-old have increased risk of AMD.

## 1. Introduction

Age-related macular degeneration (AMD) is the leading cause of blindness in the elderly population and affects 30–50 million people worldwide [1]. AMD results from a dysfunction of the retinal pigment epithelial (RPE) cell layer, which causes the accumulation of lipofuscin and drusen deposits followed by degeneration and gradual irreversible loss of high-resolution photoreceptors in the macula and impairment of the central field of vision [2,3]. The appearance of medium to large drusen deposits or pigmentary changes is prodromal and a risk factor for developing visual threatening AMD [2,4]. Late AMD includes geographic atrophy and choroidal neovascularization, with exudates [5]. To date, the precise mechanism of AMD and of its association with cardiovascular risk factors is still not fully clear [6]. Although no curative strategy for AMD is available, age-related eye disease study (AREDS) have demonstrated that a daily oral supplementation with antioxidant vitamins and minerals could reduce the risk of developing advanced AMD by 25% at 5 years in patients with drusen [7]. Smoking is reported to be greatly associated with an increasing risk of late AMD [8] and can interact with AMD gene susceptibility [9]. Smoking cessation is thus strongly advised for patients to prevent or slow the progress of AMD [10]. Thyroid hormones regulate cone opsin expression and retina development [11,12]. A recent study showed that suppressing thyroid hormone signaling could preserve cone photoreceptors in animal models of macular degeneration [13]. By contrast, administration of high doses of the thyroid hormone triiodothyronine caused cone photoreceptor death in animal models, and deletion of the thyroid hormone receptor gene could reverse this effect [14]. In a prospective cohort study, Chaker et al. discovered that higher free thyroxine levels were associated with an increased risk of AMD [15]. These studies suggested that using levothyroxine, which is also employed in thyroid suppression therapy usually administered to thyroid cancer patients, would increase the risk of AMD [10,11,12,13,14,15].

An epidemiology study showed that thyroid cancer patients have a higher risk of cardiovascular mortality [16]. Further, it is known that cardiovascular disease and AMD share common risk factors [17]. However, a population-based cohort study investigating the association between thyroid suppressive therapy and AMD in thyroid cancer patients has never been conducted. The national health insurance research database (NHIRD) contains insurance claims data of the majority of Taiwanese citizens, with a coverage rate of more than 99%, thus providing valuable information for epidemiological investigations [18]. To deeply clarify the association between thyroid suppression therapy and AMD, we used NHIRD to conduct a retrospective cohort study. We aimed to investigate whether thyroid cancer patients have a higher risk of AMD compared with age-matched controls.

## 2. Methods

### 2.1. Data Source

The single-payer national health insurance (NHI) program of Taiwan, launched in 1995, covers 99% of the 23.74 million residents of Taiwan [19]. The NHIRD contains the claims data of insurants under the NHI program. Foreigners in Taiwan are also eligible for this program. The database of this program contains registration files and original claim data for reimbursement. Large computerized databases derived from this system by the national health insurance administration (the former bureau of national health insurance, BNHI), inistry of Hhealth and welfare (the former department of health, DOH), Taiwan, and maintained by the national health research institutes, Taiwan, are provided to scientists in Taiwan for research purposes [19,20,21]. The international classification of diseases, ninth revision, clinical modification (ICD-9-CM) was used to code the diagnoses correctly. Several NHIRD-based studies of AMD and thyroid cancer have been published, thus the diagnosis of AMD and thyroid cancer in this study were reliable [22,23]. 

### 2.2. Ethics Statement

The NHIRD encrypts patients’ personal information to protect privacy and provides researchers with anonymous identification numbers associated with relevant claims information, including sex, date of birth, medical services received, and prescriptions. Therefore, patient consent is not required to access the NHIRD. This study was approved to fulfill the condition for exemption by the institutional review board (IRB) of China Medical University (CMUH104-REC2-115-CR2). The IRB also specifically waived the consent requirement.

### 2.3. Study Population

In this study, we identified patients who were older than 50 years-old and newly diagnosed with thyroid cancer (ICD-9-CM code 193) between 2000 and 2008 from the NHIRD. We extracted data of 5253 thyroid cancer patients without macular degeneration (ICD-9-CM code 362.5) before the index date. For each patient with thyroid cancer, we randomly selected four controls without a history of thyroid cancer or macular degeneration and frequency-matched them with the thyroid cancer patients by age (in 5-year spans), sex, occupation, urbanization level, and index year (non-thyroid cancer cohort).

### 2.4. Outcome and Relevant Variables

Age-related macular degeneration (ICD-9-CM code 362.5), including drusen degenerative (ICD-9-CM code 362.57), exudative senile macular degeneration (ICD-9-CM code 362.52), non-exudative macular degeneration (ICD-9-CM code 362.51), and cystoid macular degeneration (ICD-9-CM code 362.53), was defined as the endpoint of this study. The patients were followed up from the index date to the occurrence of the endpoint, withdrawal from the NHI program, or 31 December 2011. The sociodemographic variables used in this study were: age (50–64 years-old, and ≥65 years-old), occupation (white-collar workers, blue-collar workers, and other occupations), and urbanization level. The urbanization level was divided into four levels according to the NHRI report (level 1 was the highest and level 4 was the lowest urbanization level). Comorbidities at the baseline included diabetes (ICD-9-CM code 250), hypertension (ICD-9-CM codes 401–405), hyperlipidemia (ICD-9-CM code 272), coronary artery disease (CAD) (ICD-9-CM codes 410–414), high myopia (ICD-9-CM codes 367.1 and 360.21), vitreous floaters (ICD-9-CM code 379.24), congenital hyperthyroidism (ICD-9-CM code 243), acquired hypothyroidism (ICD-9-CM code 244), hyperthyroidism (ICD-9-CM code 242), asthma (ICD-9-CM code 493), chronic obstructive pulmonary disease (COPD) (ICD-9-CM codes 491, 492, 496), stroke (ICD-9-CM codes 430-438), and tobacco dependency (ICD-9-CM code 305.1). Thyroid cancer-related treatments (including radiotherapy, chemotherapy, thyroxine, and thyroidectomy) and frequency of medical visits/per year were also considered.

### 2.5. Validation

We validated the ICD-9-CM code for AMD by reviewing the chart records from the outpatient claims database in China Medical University Hospital, a 2054-bed teaching hospital in mid-Taiwan. Fifty patients were randomly selected over the period 2010 and 2014, and 10 patients were selected each year. The ICD-9 codes of this database and those of the NHIRD were used for reimbursements. AMD was established by ophthalmoscopy and fluorescence angiogram examination. A positive predictive value was estimated. Similar validation procedures were utilized in a previous study [24]. Among the randomly selected 50 outpatients coded with AMD for validation, two patients had (ICD-9 codes 362.5) with drusen deposits noted in the ophthalmoscopic exams without symptoms, thus not qualifying for (ICD-9 code 362.5). Therefore, 48 patients with AMD were confirmed by the ophthalmoscopic findings and chart review, yielding a positive predictive value of 0.96 (95% confidence interval, 0.95–1.01).

### 2.6. Statistical Analyses

The distributions of categorical and sociodemographic factors, including age, sex, urbanization level, occupation, comorbidity, and treatment, were compared between the thyroid cancer and the non-thyroid cancer cohorts. The age was matched by 5-year spans as a single category. Therefore, age was a categorical variable. To control possible confounding factors and increase the power, 1:*m* (i.e., number of study participants: number of control participants), strategy for cancer case-control study was used. Since the statistical efficiency does not gain much when *m* > 4, *m* = 4 was chosen for this study design. The differences were determined using the Chi-squared test or Mann–Whitney U test. The cohort-specific survival curves were plotted by multivariable Cox proportional hazard regression models after adjusting for comorbidities of diabetes, hypertension, hyperlipidemia, CAD, myopia, hypothyroidism, hyperthyroiodism, asthma, COPD, stroke, and tobacco dependency. The follow-up period (in person/year) was used to estimate the incidence density rates of macular degeneration according to age, sex, comorbidity, and treatment. The Cox model was used to estimate the hazard ratios (HRs) and 95% confidence intervals (CIs). By controlling for the competing risk of death, we used the Fine and Gray model, which extends the standard univariable and multivariable Cox proportional hazard regression models; to estimate the risk of macular degeneration, a proportional hazards model for the subdistribution of a competing risk was used. Furthermore, we considered propensity score matching as a sensitivity analysis for estimating the risk of macular degeneration. We also performed the sensitivity analysis which considered the time-dependent Cox regression model. All data analyses were performed using the statistical package SAS for Windows (Version 9.4, SAS Institute Inc., Carey, NC, USA). A 2-tailed *p* value of <0.05 was considered statistically significant.

## 3. Results

**Figure 1:**
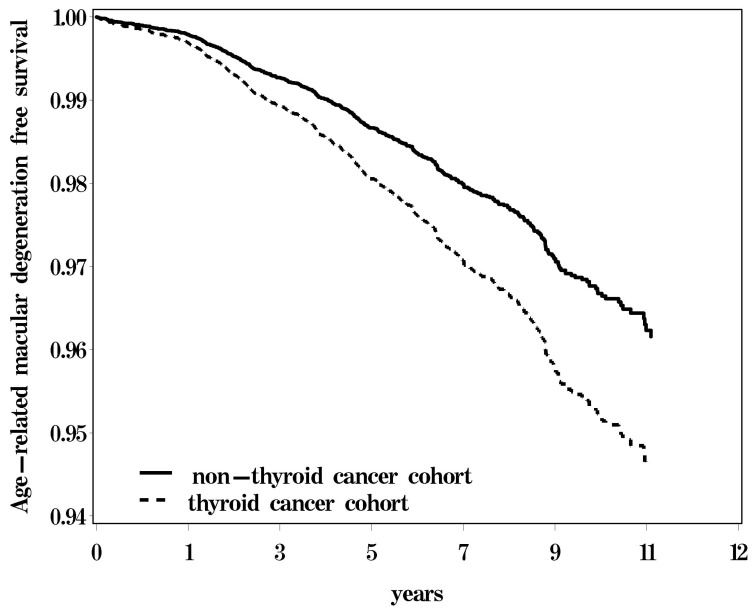
The probability of age-related macular degeneration (AMD)-free survival of the two cohorts.

This study enrolled 5253 thyroid cancer patients and 21,012 age—and gender—matched controls. Table 1 presents a comparison of the baseline characteristics of the thyroid cancer patients and of the subjects without thyroid cancer. Their respective median interquartile range (IQR) ages were 59.1 (IQR = 53.7–67.4) and 59.2 (IQR = 53.7–67.6) years for both thyroid cancer patients and controls. High proportions of the thyroid cancer and non-thyroid cancer cohorts were aged 50–64 years-old (68.3%), specifically, women (75.5%), white-collar workers (47.0%), and subjects living in urban areas (59.2%). Comorbidities were more common in the thyroid cancer cohort (*p* < 0.05), except for tobacco dependency. Figure 1 presents the 12-year cumulative incidence curves of macular degeneration by thyroid status adjusted for comorbidities. The difference in the cumulative incidence curves of macular degeneration was higher in the thyroid cancer cohort than in the non-thyroid cancer cohort.

The overall incidence densities of AMD were 27.9 and 40.7 per 10,000 person/year in the non-thyroid cancer and thyroid cancer cohorts, respectively (Table 2). The risk of AMD was significantly higher in the thyroid cancer cohort than in the non-thyroid cancer cohort (adjusted HR = 1.38, 95% CI = 1.09–1.75) (Table 3). The male patients with thyroid cancer had a significant higher risk of AMD than the male patients without thyroid cancer. The risk of AMD was higher in patients with comorbidities in the thyroid cancer cohort than in those with comorbidities in the non-thyroid cancer cohort. After adjustment for all relevant confounding factors in the competing risk regression model, the risk of AMD remained significantly higher in the thyroid cancer cohort than in the non-thyroid cancer cohort (adjusted SHR = 1.57, 95% CI = 1.26–1.97) (Table 4). These patients received partial thyroidectomy and did not need thyroid suppression therapy. Thyroid cancer patients receiving levothyroxine had a non-significantly higher risk of AMD compared with thyroid cancer patients not receiving levothyroxine (adjusted SHR = 0.85, 95% CI = 0.59–1.22). The cumulative censoring rate over 12 years (2000–2011) was 18.9% in the thyroid cancer cohort, which was higher than that in the non-thyroid cohort (14.1%). The possible reasons for the discontinuity of national health insurance include death, withdrawal of insurance, immigration, prison sentence, etc.

The patients with thyroid cancer had still a significant higher risk of AMD than the non-thyroid cancer cohort subjects in the propensity score matching (adjusted HR = 1.48, 95% CI = 1.12–1.97) (Table 5).

## 4. Discussion

Although associations of thyroid hormone levels with AMD have been observed in animal models and epidemiological research, whether thyroid cancer has an effect on the risk of AMD is unknown. This was the first retrospective largest cohort study reporting that thyroid cancer patients exhibited a 1.38-fold higher risk of AMD than control subjects. Receiving levothyroxine would not increase the risk of AMD in thyroid cancer patients. 

Our finding of the association of thyroid cancer with the risk of AMD has several explanations. First, thyroid cancer and AMD have some common etiologies, including hyperinsulinemia state, obesity, and stimulation of thyroid growth. Obesity and hyperinsulinemia state have been linked to an increased risk of thyroid cancer [25,26]. Several cross-sectional and longitudinal studies have also reported a close association between central obesity and AMD [27,28,29]. The POLA study and age-related eye disease study have reported that obesity patients had a significant increased risk of late AMD, pigmentary abnormalities, and AMD progression [17,30]. AMD has been hypothesized to have a common CAD risk profile, including hyperinsulinemia [31]. Though the mechanism of the association of hyperinsulinemia and AMD is not fully elucidated, Samules et al. have discovered an altered RPE function concomitant with the onset of hyperglycemia in mouse models [32]. Further, the RPE dysfunction was reversible and mitigated in hyperinsulinemic B6.BKS.Lepr mice [32]. Considering that our thyroid cancer patients had a higher prevalence of diabetes, hypertension, hyperlipidemia, and CAD, we suggest that the hyperinsulinemia state and oxidative stress cause the development of thyroid cancer as well as of RPE dysfunction and AMD [33,34]. This would explain why the thyroid cancer patients here examined had increased risk of AMD. Second, mitochondrial dysfunction would be another mechanism for higher risks of AMD in thyroid cancer patients. Mitochondrial dysfunction plays a common role in metabolic diseases, degenerative disease, and cancer [35]. Tumor-specific of somatic mitochondrial DNA mutations have been identified in thyroid tumors [36]. 

Mitochondrial DNA variants, i.e., haplogroups J and U, have been reported to be associated with risks of increasing drusen, retina pigment abnormalities, and macular degenerations [37]. Further studies are necessary to investigate whether thyroid cancer and AMD have common pathogenic pathways involving genetic or somatic mutations. It will be important to clarify to what extent these two diseases could be reversed or modulated.

The age-related eye disease study and beaver dam eye study have both reported an association between thyroid hormone use and AMD [38,39]. Chaker et al. reported that higher free thyroxine levels were associated with an increased risk of AMD [15]. It has been recognized that subclinical hyperthyroidism is often observed in thyroid cancer patients receiving levothyroxine, occasionally complicated by arrhythmia and osteoporotic fracture [40,41]. Thus, shortening the time period of thyroid-stimulating hormone (TSH) suppression or limiting the levothyroxine dosage is always a goal in treating thyroid cancer patients. Our data show a positive aspect of levothyroxine use in thyroid cancer patients, indicating that levothyroxine is not associated a with higher risk of AMD in thyroid cancer patients, consistent with the findings of Güveli et al. [42]. 

Aging is a key risk factor for AMD and thyroid cancer in the general population. A study reported that AMD is most prevalent within the 50–65 age group in the general population [19]. In this study, after stratification by age, thyroid cancer patients aged ≥65 years-old exhibited a higher adjusted risk (1.52-fold) compared with the controls across all age stratifications. This association is particularly of concern for the elderly, warrantying close monitoring of vision impairment and retina conditions. Our data also showed that thyroid cancer patients, especially those with comorbidities and male patients, had higher risk of AMD, suggesting that comorbidities and male sex may contribute to the development of AMD. Although a clear association between the risk of AMD and gender has not been determined, clinical attention is warranted for these specific groups. 

This study has several strengths. First, the NHIRD contains the medical claims data of >22 million people in Taiwan, with a coverage rate of >98%, thus providing a large representative sample for clinical investigation. The National Health Insurance Administration strictly monitors and checks the insurance claims for repayment to prevent healthcare fraud; hence, the diagnosis and procedure codes in the NHIRD are reliable. Therefore, although some potential risk factors for macular degeneration, including daily antioxidants intake, body mass index, myopia degrees, and thyroid hormone levels, are unavailable in the NHIRD, we believe that we have considered the effects of these unknown variables by considering diabetes, hypertension, hyperlipidemia, CAD, myopia, hyperthyroidism, and hypothyroidism. Since smoking is the major environmental risk factor for AMD [8], we have considered the comorbidities including CDPD, stroke, asthma, and tobacco dependency as the proxies for smoking habits. A similar strategy accounting for this unavailable smoking variable in the NHIRD has been used in another study [43]. After adjusting for these proxies of smoking, thyroid cancer patients still had a 1.67-fold higher risk of AMD, suggesting thyroid cancer is a risk factor for AMD. Furthermore, although smoking is a common risk factor for many cancers, including lung cancer [44], oral cancer [45], esophageal cancer [46], etc., several studies have found that smoking is protective for thyroid cancer. Smoking would decrease the simulation of TSH and estrogen in thyroid tissues, thus lessening the risk of thyroid cancer [47]. It is also reported that a genetic variant is the cause of the inverse relationship between smoking and thyroid cancer [48]. The majority of thyroid cancer patients are women. In our study, about 75% of our thyroid cancer participants were women. Asian women have been reported to have a relatively lower prevalence of smoking (less than 7%) than men [49]. Therefore, taken these study results together, it is reasonable to assume that a possible bias resulting from missing the smoking variable would be subtle in this study. Finally, since thyroid cancer patients with active disease underwent more medical visits and ophthalmological examinations, we have adjusted the frequency of medical visits per year to avoid a possible surveillance bias in this study.

This study has some limitations. We did not have direct information on the presence of single nucleotide polymorphisms (SNP) for each patient. Considering the relatively small sample size of this study, the possibility of it being underpowered might occur. Adding the SNP information (e.g., complement H) to the multivariate model might also alter the risk estimate of AMD in thyroid cancer. This study is a nationwide cohort investigation including 5253 thyroid cancer patients and a follow-up of 12 years. The age-standardized rate of thyroid cancer in our study population is comparable to that of Asian countries and of the worldwide population [50,51]. Hence, our study findings exhibit a more favorable generalizability than cross-sectional studies with small samples. Finally, we did not have information about tumor staging, pathological findings, and lymph node involvement for each thyroid cancer patients. Possibly, under-diagnosed, underestimated, asymptomatic, or mild AMD were other study limitations. The information about thyroid hormone levels was unavailable in NHIRD. Thus, we could not correlate the disease occurrence with thyroid hormone levels.

## 5. Conclusions

The current study determines that thyroid cancer is associated with an increased risk of AMD. Our data suggest that clinical awareness should be maintained by close monitoring for the possible prevention of AMD development in thyroid cancer patients.

## Figures and Tables

**Table 1:** Demographic factors and comorbidities of the study participants according to their thyroid cancer status.

	Control *N* = 21,012		Thyroid Cancer *N* = 5253		*p*-Value *
Variable	*n*	%	*n*	%	
**Age, years**					0.99
50–64	14,356	68.3	3589	68.3	
≥65	6656	31.7	1664	31.7	
Median (IQR) ^†^	59.2	(53.7–67.6)	59.1	(53.7–67.4)	0.13
**Sex**					0.99
Female	15856	75.5	3964	75.5	
Male	5156	24.5	1289	24.5	
**Occupation**					0.99
White-collar	9876	47.0	2469	47.0	
Blue-collar	9712	46.2	2428	46.2	
Others	1424	6.78	356	6.78	
**Urbanization level ^§^**					0.99
1 (highest)	6172	29.4	1543	29.4	
2	6264	29.8	1566	29.8	
3	3400	16.2	850	16.2	
4 (lowest)	5176	24.6	1294	24.6	
Frequency of medical visits/per year	5.47	13.5	22.3	17.7	<0.001
**Comorbidity**					
Diabetes	2949	14.0	938	17.9	<0.001
Hypertension	9600	45.7	2829	53.9	<0.001
Hyperlipidemia	6262	29.8	1718	32.7	<0.001
CAD	4618	22.0	1227	23.4	0.03
High myopia	116	0.55	47	0.89	0.005
Hypothyroidism	216	1.03	812	15.5	<0.001
Hyperthyroidism	506	2.41	47	0.89	<0.001
Asthma	1688	8.03	516	9.82	<0.001
COPD	2581	12.3	874	16.6	<0.001
Stroke	996	4.74	212	4.04	0.03
Tobacco dependency	74	0.35	16	0.30	0.09
**Treatment**					
Radiotherapy			629	12.0	
Chemotherapy			331	6.30	
Thyroxin			3536	67.3	
Thyroidectomy			4077	77.6	
I-131			3466	66.0	

* Comparison between thyroid cancer and control using the Chi-square test for categorical variables and the *t* test for continuous variables, respectively. ^†^ Mann–Whitney U test; ^§^ The urbanization level was categorized by the population density of the residential area into four levels, with level 1 as the most urbanized and level 4 as the least urbanized. Other occupations included primarily retired, unemployed, or low-income populations. CAD: coronary artery disease, COPD: chronic obstructive pulmonary disease.

**Table 2:** Crude and adjusted hazard for macular degeneration in the thyroid cancer and control groups.

	Control (*N* = 21,012)	Thyroid Cancer (*N* = 5253)
**Macular degeneration**		
Number	372	126
persons/year	133,311	30,927
Incidence rates	27.9	40.7
Crude HR (95% CI)	1.00	1.47 (1.20, 1.80) ***
Adjusted HR (95% CI) ^‡^	1.00	1.38 (1.09, 1.75) **
**Drusen degenerative**		
Number	15	8
Incidence rates	1.13	2.59
Crude HR (95% CI)	1.00	2.28 (0.96, 5.37)
Adjusted HR (95% CI) ^‡^	1.00	2.05 (0.75, 5.58)
**Exudative senile macular degeneration**		
Number	29	7
Incidence rates	2.18	2.26
Crude HR (95% CI)	1.00	1.05 (0.46, 2.40)
Adjusted HR (95% CI) ^‡^	1.00	0.93 (0.36, 2.38)
**Non-exudative macular degeneration**		
Number	33	14
Incidence rates	2.48	4.53
Crude HR (95% CI)	1.00	1.84 (0.98, 3.43)
Adjusted HR (95% CI) ^‡^	1.00	1.88 (0.89, 3.98)
**Cystoid macular degeneration**		
Number	35	11
Incidence rates	2.63	3.56
Crude HR (95% CI)	1.00	1.36 (0.69, 2.67)
Adjusted HR (95% CI) ^‡^	1.00	1.33 (0.60, 2.96)

Abbreviation: IR, incidence density rates, per 10,000 person/year; HR, hazard ratio; CI, confidence interval; ^‡^ adjusted for age and frequency of medical visits/per year for comorbidities, i.e., diabetes, hypertension, hyperlipidemia, CAD, myopia, hypothyroidism, hyperthyroiodism, asthma, COPD, stroke, and tobacco dependency; ** *p* < 0.01, *** *p* < 0.001.

**Table 3:** Adjusted hazard ratios (HR) for macular degeneration according to thyroid cancer status stratified by age, sex, and comorbidity.

	Control	Thyroid Cancer
		Adjusted HR ^†^ (95% CI)
**Age**		
50–64	1.00	1.33 (0.96, 1.82)
≥65	1.00	1.36 (0.95, 1.94)
**Sex**		
Female	1.00	1.26 (0.96, 1.65)
Male	1.00	1.92 (1.18, 3.14) **
**Comorbidity**		
No	1.00	0.75 (0.37, 1.53)
Yes	1.00	1.38 (1.09, 1.74) **

Abbreviation: HR, hazard ratio; CI, confidence interval. ^†^ Adjusted for age and frequency of medical visits/per year for comorbidities, i.e., diabetes, hypertension, hyperlipidemia, CAD, myopia, hypothyroidism, hyperthyroiodism, asthma, COPD, stroke, and tobacco dependency; ** *p* < 0.01.

**Table 4:** Incidence and subhazard ratio (SHR) of macular degeneration in patients with thyroid cancer compared to those without thyroid cancer using the competing-risks regression models.

	Competing-Risks Regression Models	
	Thyroid Cancer	
	No	Yes
	(*N* = 53,784)	(*N* = 13,446)
Crude SHR (95% CI)	1 (Reference)	1.76 (1.44, 2.1 6) ***
Adjusted SHR ^†^ (95% CI)	1 (Reference)	1.57 (1.26, 1.97) ***

Crude SHR, relative subhazard ratio; Adjusted SHR ^†^: multivariable analysis including age, frequency of medical visits/per year for comorbidities, i.e., diabetes, hypertension, hyperlipidemia, CAD, myopia, hypothyroidism, hyperthyroiodism, asthma, COPD, stroke, and tobacco dependency. *** *p* < 0.001

**Table 5:** Incidence and hazard ratio of macular degeneration in patients with thyroid cancer compared to subjects without thyroid cancer by propensity score-based sensitivity analysis.

	Control	Thyroid Cancer
	(*N* = 4612)	(*N* = 4612)
Persons/year	29,107	27,078
Macular Degeneration		
Event	83	110
Rate	28.5	40.6
Crude HR (95% CI)	1.00	1.43 (1.08, 1.90) *
Adjusted HR (95% CI) ^‡^	1.00	1.48 (1.12, 1.97) **

Abbreviation: IR, incidence density rates, per 10,000 persons/year; HR, hazard ratio; CI, confidence interval; ^‡^ adjusted for age, frequency of medical visits/per year for comorbidities, i.e., diabetes, hypertension, hyperlipidemia, CAD, myopia, hypothyroidism, hyperthyroiodism, asthma, COPD, stroke, and tobacco dependency; * *p* < 0.05, ** *p* < 0.01.

## Abbreviations

AMD	age-related macular degeneration
CI	confidence interval
HR	hazard ratio
ICD-9-CM	international classification of diseases, ninth revision, clinical modification
IQR	interquartile range
NHIRD	national health insurance research
